# Supplementary material for: A UMLS-based spell checker for natural language processing in vaccine safety
Source: BMC Med Inform Decis Mak. 2007 Feb 12;7:3. doi: 10.1186/1472-6947-7-3 (PMC1805499; doi:10.1186/1472-6947-7-3)
Supplement: Additional file 13 — RAP application directory. Sets up directory for RAP (RDF application for PHP) source code [file 1472-6947-7-3-S13.gz › rap/doc/phpdoc/__filesource/fsource_dataset__datasetIteratorAllGraphsDb.php.html]

xml version="1.0" encoding="iso-8859-1"?


File Source for IteratorAllGraphsDb.php


# Source for file IteratorAllGraphsDb.php

Documentation is available at IteratorAllGraphsDb.php

```
1. <?php
2. /**
3. * ----------------------------------------------------------------------------------
4. * Class: IteratorAllGraphsDb
5. * ----------------------------------------------------------------------------------
6. *
7. * @package     dataset
8. */


11. /**
12. * Implementation of a Graph iterator.
13. *
14. * This Iterator should be used in a for-loop like:
15. * for($iterator = $dataset->listGraphs(); $iterator->valid(); $iterator->next())
16. * {
17. *    $currentResource=$iterator->current();
18. * };
19. *
20. * <BR><BR>History:
21. * <LI>05-03-2005                : First version of this class.</LI>
22. *
23. * @version  V0.9.1
24. * @author Daniel Westphal <mail at d-westphal dot de>
25. *
26. *
27. * @package     dataset
28. * @access    public
29. ***/
30. class IteratorAllGraphsDb
31. {
32. /**
33. * Holds a reference to the associated DB resultSet
34. * @var        $dbResultSets ADODB result
35. * @access    private
36. */
37. var $dbResultSet;

39. /**
40. * Holds a reference to the associated datasetDb
41. * @var        datasetDb
42. * @access    private
43. */
44. var $datasetDb;


47. /**
48. * The current position
49. * @var        integer
50. * @access    private
51. */
52. var $key;


55. /**
56. * The current NamedGraph
57. * @var obejct NamedGraph
58. * @access    private
59. */
60. var $current;


64. /**
65. * Constructor.
66. *
67. *
68. * @param ADODBResultSet
69. * @param DatasetDb
70. * @access    public
71. */
72. function IteratorAllGraphsDb(&$dbResultSet,&$datasetDb)
73. {
74. $this->dbResultSet=& $dbResultSet;
75. $this->datasetDb=& $datasetDb;
76. $this->current = $this->dbResultSet->fields[0];
77. }

79. /**
80. * Resets iterator list to start
81. *
82. * @access public
83. */
84. function rewind()
85. {
86. //not supported
87. }

89. /**
90. * Says if there are additional items left in the list
91. *
92. * @return    boolean
93. * @access    public
94. */
95. function valid()
96. {
97. return (!$this->dbResultSet->EOF);
98. }

100. /**
101. * Moves Iterator to the next item in the list
102. *
103. * @access    public
104. */
105. function next()
106. {
107. $this->dbResultSet->moveNext();
108. $this->current = $this->dbResultSet->fields[0];
109. }

111. /**
112. * Returns the current item
113. *
114. * @return    mixed
115. * @access    public
116. */
117. function &current()
118. {
119. return ($this->datasetDb->getNamedGraph($this->current));
120. }


123. /**
124. * Returns the key of the current item
125. *
126. * @return    integer
127. * @access    public
128. */
129. function key()
130. {
131. return $this->dbResultSet->_currentRow;
132. }
133. }
134. ?>
```

Documentation generated on Thu, 7 Jul 2005 13:41:45 +0200 by phpDocumentor 1.3.0RC3
